# International practices in health technology assessment and public financing of digital health technologies: recommendations for Hungary

**DOI:** 10.3389/fpubh.2023.1197949

**Published:** 2023-08-31

**Authors:** Fruzsina Mezei, Krisztián Horváth, Máté Pálfi, Kornélia Lovas, Ildikó Ádám, Gergő Túri

**Affiliations:** ^1^Data-Driven Health Division of National Laboratory for Health Security, Health Services Management Training Centre, Semmelweis University, Budapest, Hungary; ^2^EIT Health France, Paris, France; ^3^Department of Public Health, Semmelweis University, Budapest, Hungary; ^4^Center for Health Technology Assessment, Semmelweis University, Budapest, Hungary; ^5^CE Certiso Ltd, Budakeszi, Hungary; ^6^Department of Clinical Pharmacy, University of Szeged, Szeged, Hungary; ^7^Epidemiology and Surveillance Centre, Semmelweis University, Budapest, Hungary; ^8^Synthesis Health Research Foundation, Budapest, Hungary

**Keywords:** digital health technologies, digital health, eHealth, mHealth, reimbursement, HTA, financing, Hungary

## Abstract

**Background:**

Evaluating and integrating digital health technologies is a critical component of a national healthcare ecosystem in the 2020s and is expected to even increase in significance.

**Design:**

The paper gives an overview of international practices on public financing and health technology assessment of digital health technologies (DHTs) in five European Union (EU) countries and outlines recommendations for country-level action that relevant stakeholders can consider in order to support uptake of digital health solutions in Hungary. A scoping review was carried out to identify and gather country-specific classifications and international practices on the financing DHTs in five pioneering EU countries: Germany, France, Belgium, the United Kingdom and Finland.

**Results:**

Several frameworks have been developed for DHTs, however there is no single, unified framework or method for classification, evaluation, and financing of digital health technologies in European context. European countries apply different taxonomy, use different assessment domains and regulations for the reimbursement of DHTs. The Working Group of the Hungarian Health Economic Society recommends eight specific points for stakeholders, importantly taking active role in shaping common clinical evidence standards and technical quality criteria across in order for common standards to be developed in the European Union single market.

**Conclusion:**

Specificities of national healthcare contexts must be taken into account in decisions to allocate public funds to certain therapies rather than others.

## 1. Introduction

Looking beyond the COVID-19 pandemic and its consequences, today is a historic opportunity to make digital technologies an integral part of public health service. Digital health technologies (DHTs) have been rapidly proliferating in recent years to meet the growing demand for innovative forms of healthcare solutions, at increasingly competitive cost. Advances in large language models (LLM) are enabling natural language algorithms to automatically process clinical records and transform into structured data, that can serve as input for clinical decision support systems (1). Deep Learning algorithms are revolutionizing radiology and oncology by enabling auto-segmentation of cancer lesions, reaching parity in accuracy with trained radiologist, at a much faster pace and lower cost, enabling physicians to focus on the most complex cases. We are witnessing the emergence of use-cases across the specialties of healthcare driven by private sector innovation, yet EU regulators are often caught one step behind the curve. With the implementation of appropriate, well-tailored digital health strategy, the digital transformation of healthcare has the potential to be disruptive, with more equitable and accessible care for all European citizens and offer greater personalization and value to the individual patient. The realization of these benefits however requires close collaboration between the health industry and regulatory bodies, and a uniform framework for evaluation and reimbursement, as monetization is a key factor in driving innovation, the main component of competitiveness in a developed economy.

Health Technology Assessment (HTA) is one of the key prerequisites for public funding of health technologies in EU countries. HTA is difficult to standardize even in the case of pharmaceuticals and (non-DHT) medical devices, where decades of experience are available, with well-developed methodologies. The questions around methodologies regarding the appraisal of DHTs are of significantly greater magnitude as they cover such a broad range of technologies and use-cases, that makes the development of standardized processes very challenging. The traditional clinical domains of HTA (relative safety, relative clinical effectiveness) may not be suited in every case of DHTs, highlighting the need to update or develop specific methodological framework with new elements such as data privacy, interoperability, usability and different outcome categories to measure added value, while keeping patient safety always at the forefront (2, 3).

To make digital health applications and platforms more accessible, several European countries are in the process of adopting a statutory reimbursement obligation by amending the traditional assessment frameworks, to fit the particularities of digital health solutions. Looking at reimbursement process of EU countries, different regulations can be identified for DHTs, while there is clearly an increased interest in implementing reimbursement options coupled with assessment frameworks (4, 5). The reimbursement options are mixed: in some countries DHTs are partly be paid voluntarily by patients and individual health insurers, in others, a DHT with certain proof of patient benefit are paid obligatorily by all health insurers (e.g., Germany) (6). The view of the authors is that DHTs should reflect a care process rather than a single product, warranting a different approach than “traditional” HTA. The principles underpinning decisions on their reimbursement should be comparable, as DHTs compete for public fundings as well.

Value-based evaluation is crucial for the integration of DHTs into the healthcare systems and the sustainability of the innovation sphere, however (patient) value is anything but straightforward to determine. Approaches for demonstrating the benefit of DHTs are increasingly emerging in Europe that aim to consider the particular characteristics of these health technologies, gathering evidence (quality and quantity) to demonstrate whether the solution is superior to the current standard of care, or fit for filling the gaps of traditional care. Examples of countries with DHT assessment frameworks include Belgium, the United Kingdom, Germany, France and Finland, with Germany being considered a pioneer, having already introduced a statutory reimbursement obligation for patient-facing digital health apps and platforms or “DiGAs” (Digitale Gesundheitsanwendungen) (7–11). Despite the considerable progress made by some EU countries, many Member States do not have clear regulatory frameworks and funding mechanisms to distinguish digital therapeutics from the abundance of available wellness, mHealth apps. While the pioneering countries can serve as an example (with their own shortcomings), low-and middle-income countries are still in need of methodological support on how to value, reimburse and facilitate the uptake of DHTs, and must navigate DHT transformation carefully, as the available resources are limited, thus the opportunity cost of reimbursement decisions is greater, compared to more affluent countries.

Our study examines 6 EU countries in terms of DHT framework, but focuses on Hungary, a middle-income country in Central – Europe with a population of 9.7 million and a GDP *per capita* of less than 40% compared to Austria, that could significantly benefit, in our view, from methodological support. Hungary’s healthcare system is funded by tax and social health insurance contribution revenues and organized by a single-payer, the National Health Insurance Fund (NHIF) (12). The country’s Office of Health Technology Assessment (OHTA) was established in 2004 to review proposals for reimbursement for various health technologies, such as pharmaceuticals, medical devices and other medical technologies (13). The OHTA is currently part of the National Institute of Pharmacy and Nutrition and prepares the clinical and economic evaluation of health technologies based on the current health economic guideline in Hungary (14). The Health Technology Assessment Committee, part of the NHIF, recommends reimbursement of specific health technologies based on the assessment of the OHTA and the recommendation of the relevant College of Medical Professionals (15). The Director General of the NHIF makes the final decision on public funding of a certain technology, but in some cases the Ministry of the Interior is also involved in the decision-making. There are currently no established practices and guidelines for the HTA of DHTs in Hungary.

The Hungarian Health Economics Society has launched a working group to overview existing international practices for HTA of digital health technologies and assess potential implications for current practices of HTA in Hungary. The primary objectives of the working group were as follows:

- To identify and review existing frameworks for classification of digital health technologies in the European Union,
- To overview the international practices on evaluation and public financing of digital health technologies,

Secondary objectives pursued were to outline a set of recommendations for country-level action for Hungary, − based on international practices of assessment and reimbursement of digital health technologies - that relevant stakeholders can consider in order to support uptake of digital health solutions in Hungary.

The findings reported in this article are published on behalf of the Digital Health working group of the Hungarian Health Economic Society. Authors did not receive compensation for their contribution in the study, all activities were carried out *pro bono*.

## 2. Methods

A scoping review was carried out in July 2023 to overview country-specific classifications of digital health technologies (focusing on EU) and international practices on public financing and HTA of digital health technologies. The review was performed according to the PRISMA guideline (16). The study protocol specified the main objectives of the study, the search strategy, the eligibility criteria, the selection of sources of evidence, and the method of the analysis.

The literature search was performed on PubMed, Google Scholar and Embase databases using the following keywords: digital health or digital health technologies or digital health technology or digital health application or e-health or ehealth or mhealth or m-health and financing or finance or health technology assessment or reimbursement or public reimbursement or HTA. In addition, the literature search collected information on the classification framework and financing of digital health technologies in 5 countries: Germany, France, Belgium, the United Kingdom and Finland. The literature review had been limited to these European countries as they had well-developed processes for introducing and regulating digital health technologies, and several publications and reports describing these systems can be identified, thus we considered them “pioneering EU countries.” Peer-reviewed journal papers and gray literature were included if they were published between 2013 and 2023, written in German, English, French or Hungarian, and contained relevant information on the public financing of digital health technologies. While collecting grey literature, reports and documents prepared by government institutions and agencies, international professional organizations, and academic centers were identified.

**Figure 1:**
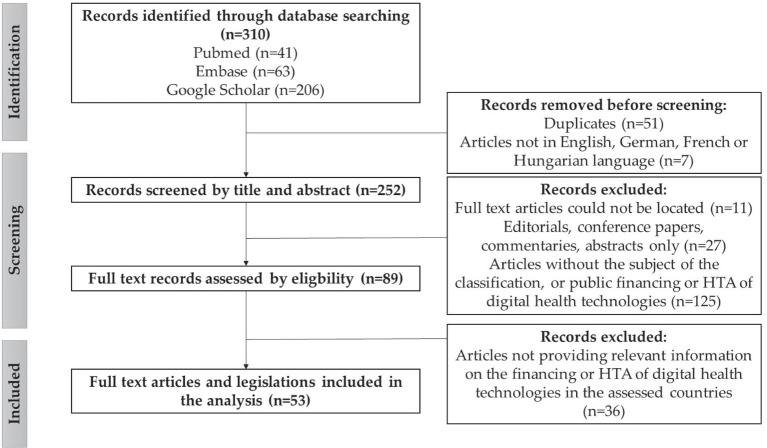
Flowchart of the literature search results on the international practices on public financing and HTA of digital health technologies, according to the PRISMA guideline (16). Source: authors, based on the PRISMA guideline (16).

Exclusion criteria were publications published before 2013, editorials, conference papers, commentaries, abstract only publications, and did not contain specific and relevant information on the classification of digital health technologies or the international practices of the reimbursement and health technology assessment of digital health technologies of the countries under review. The literature search resulted in 310 publications; of which 53 were included in the final analysis (Figure 1).

Each reviewer screened the publications identified in the literature search and, working in pairs, assessed the title, abstract, and then the full text of potentially relevant publications in the review. In case of disagreement, a third reviewer was involved. Publications were organized according to a coding frame with label definitions using Atlas.ti software. Three research team members did the coding in parallel, and the entire team interpreted the results and formulated recommendations for Hungary. The HTA practices of digital health technologies of the countries under review were assessed according to the following five criteria: the assessment framework, the public reimbursement; the types of technologies funded; the assessment criteria; and the clinical evidence criteria.

**Figure 2:**
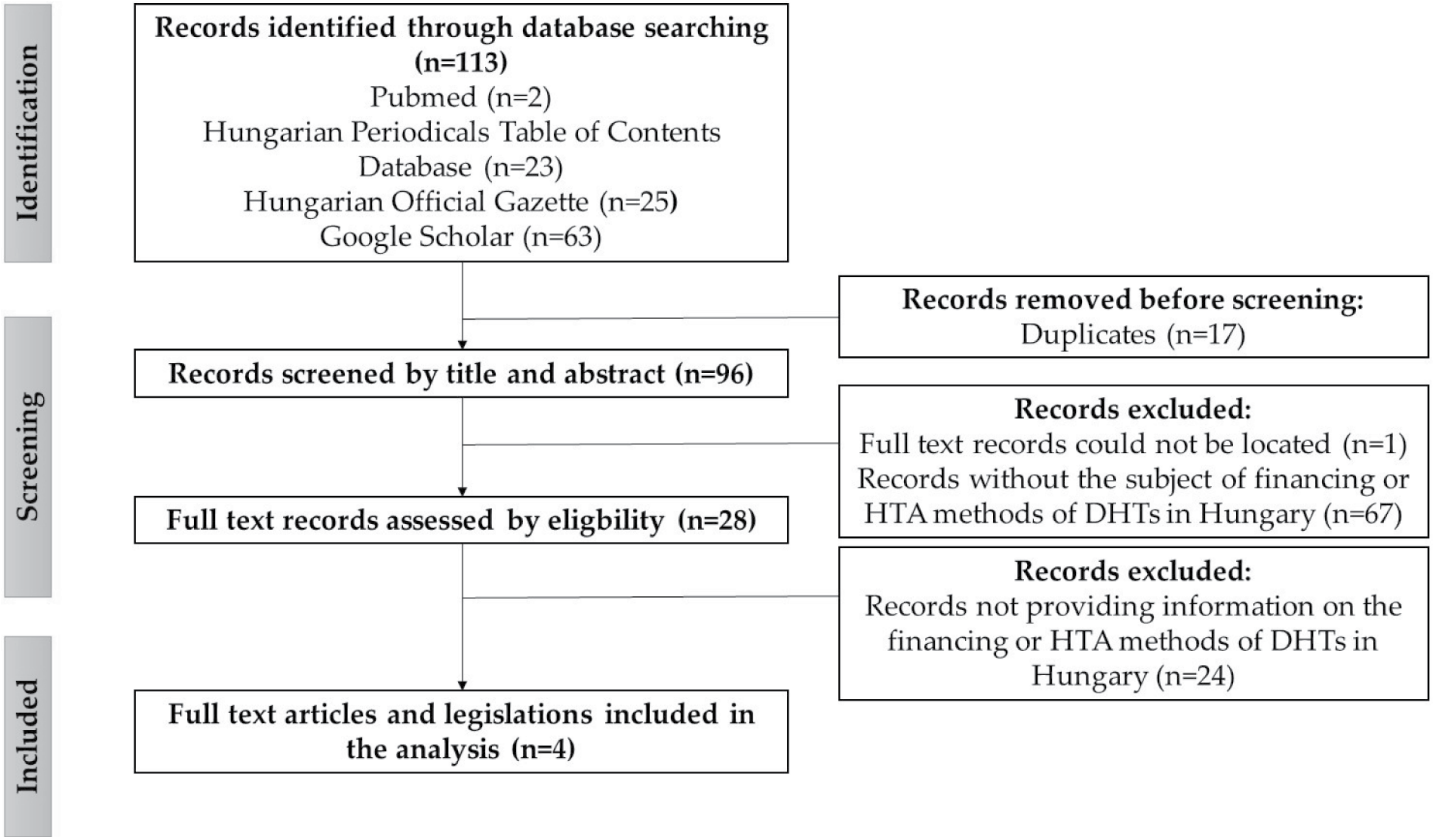
Flowchart of the literature search results on the current practice of HTA and financing of DHTs in Hungary, according to the PRISMA guideline (16). Source: authors, based on the PRISMA guideline (16).

In order to describe the current practice of technology assessment and financing of DHTs in Hungary, another literature review was conducted. The literature search was performed on PubMed, the Hungarian Official Gazette, the Hungarian Periodicals Table of Contents Database, and Google Scholar databases using the Hungarian equivalents of the above-mentioned keywords. Peer-reviewed scientific publications in English and Hungarian and current legislation in Hungarian on the topic were assessed. Exclusion criteria were publications published before 2013, editorials, conference papers, commentaries, abstract only publications, and did not contain specific and relevant information on the financing or HTA methods of digital health technologies in Hungary. The literature search resulted in 113 publications; of which 4 were included in the analysis (Figure 2).

Based on a European outlook and a review of the current Hungarian practices, the Digital Health working group of the Hungarian Health Economic Society proposed actions to support the uptake of evidence-based digital health technologies in Hungary.

## 3. Results

### 3.1. International frameworks for classification of digital health technologies

As innovation in the digital health sector accelerates, we can see diverse use cases appearing in an increasing number of therapeutic fields, ranging from preventive services to remote monitoring, and more complex decision-support platforms. The definition of digital health technologies as a collective term (digital health, telehealth, eHealth, mHealth, artificial intelligence AI–driven solutions) differs globally, and the EU member states use different nomenclature as well. The World Health Organization (WHO) defines eHeatlh as “cost-effective and secure use of information and communications technologies in support of health and health-related fields, including health-care services, health surveillance, health literature, and health education, knowledge and research” (17). Digital health and care refers to tools and services that use information and communication technologies (ICTs) to improve prevention, diagnosis, treatment, monitoring and management of health-related issues and to monitor and manage lifestyle-habits that impact health (18).

Comprehensive reviews of the definitions have been undertaken in the past, however there is still confusion over the terms used, which are sometimes used interchangeably, and even a single term may have multiple definitions (19–22).

Digital health technologies cover a wide range of tools and technologies, and the nomenclatures used can be overlapping, confusing even, as they encompass different functions, both in and outside of clinical setting. Given the heterogeneity of DHTs it is necessary to create a taxonomy that facilitates reimbursement, pricing, and prescribing decisions (4). The classification of DHTs has not yet developed into an internationally standardized and accepted practice, as is the case for pharmaceuticals or medical devices. The frameworks for the classification of DHTs in the international literature define and categorize these new technologies in different ways (23–26).

In addition, the existing DHT assessment frameworks differ in terms of the scope of DHTs they apply to. While the assessment frameworks developed by NICE and FINNCHTA look at a relatively large pool of DHTs (from smartphone apps, standalone software, robotics and AI), frameworks in Germany limit assessment to patient-facing applications and web-based platforms that are CE-marked and are risk class I or IIa. Other countries (e.g., France) are in between, opening the scope to patient and healthcare professionals facing DHTs, like telemonitoring solutions.

There is a general interest across countries to embrace and reimburse digital technologies which creates a need to understand which aspects of technology are being used, in what manner, and with what impact on health, patient safety and associated outcomes. The heterogeneous nature of DHTs, coupled with the different structures and regulations in European Union member states, adds to the complexity that DHT manufacturers face when seeking market access. Providing evidence about DHT effectiveness, safety and applicability is challenging, and we believe a joint understanding of a framework is lacking for appraisal, that can inform inclusion decision and justify public reimbursement. On a global scale this presents a hindrance for manufacturers aiming to bring DHTs to market in the EU single market, compared to US counterparts, where they can benefit from a well-developed and uniformly regulated market. Thus, the clear conceptual definition and conceptual coverage of digital health, electronic health (eHealth), mobile health (mHealth), or telehealth/telemedicine terms should be established for health outcomes research to facilitate a more precise and effective interdisciplinary communication of evidence and allow for a better assessment of these technologies.

### 3.2. International practices of HTA and public financing of digital health technologies

There is a high degree of heterogeneity in the health technology assessment frameworks for DHTs, with the 2020 study by Vis et al. identifying 23 frameworks across countries (11). According to the analysis, HTA frameworks of DHTs assess new technologies in different dimensions (such as technological, clinical, economic, legal, ethical, and organizational) and with different numbers and types of criteria. While some HTA frameworks differentiate between stages of technology development, others assess technologies at varying stages of development along the exact dimensions and criteria. The diversity of HTA frameworks for DHTs may be due to the complexity of assessing the rapidly evolving DHTs using very different technologies, which also significantly transform health and social systems and behaviors.

The monetization of DHTs is challenging, as shown by a research paper published this year, as digital health revenue accounts for only 2% of global healthcare spending. The fragmented regulatory landscape in Europe contributes to the lag of DHT uptake, as digital health stakeholders ranked Germany second and United Kingdom third, after the US as potential targets for market access. Even in Germany, the leading EU country in DHT inclusion into public reimbursement, DiGA revenues account for 0.01% of the total healthcare spending. When it comes form of reimbursement, a subscription-based payment method is considered best by DHT providers and innovators, and health plans as the preferred payors (27, 28).

In an effort to better integrate an expanding offering of digital solutions in healthcare services, a number of countries have begun to adapt their national frameworks and policies in recent years. Significant differences can be observed in the types of technologies countries have included, as well as the assessment criteria chosen to assess and make them available to patients. While Germany and Belgium are working with a smaller scope (CE-marked, low-risk health apps and web-based platforms), other countries like France, Finland and the UK are also integrating telemedicine, AI solutions, and robotics into their frameworks. Differences regarding requirements and assessment domains can also be observed, similarly to the tendency to connect the HTA to public reimbursement or not. Table 1 summarizes the results detailed in the country-specific sections below.

**Table 1:** Pioneer countries and their practices in the HTA and reimbursement of digital health technologies.

Country	Assessment framework	Types of technologies assessed	Assessment criteria	Public reimbursement (yes/no)
Belgium	mHealthBelgium validation pyramid	Mobile applications that are CE-marked as a medical device	Requirements for each level: M1: CE marking, notification to FAMHP, GDPR declaration M2: level 1 criteria, authentication, security, use of local e-health services, risk assessment M3: proven social-economic value	Yes
Finland	Digital Health Care Services/ Digi-HTA	mhealth, AI, robotics	9 assessment domains: (1) product information; (2) technical stability; (3) cost; (4) effectiveness; (5) clinical safety; (6) data security and protection; (7) usability and accessibility; (8) interoperability; (9) patient and organizational considerations.	No
France	Digital medical devices (DMDs) including telemonitoring and telemedicine	CMDs (Connected Medical Devices) which are: intended for use for medical purposes CE marked for individual use (implanted or used by patient themselves) submitted application for reimbursement.	CE marked, provide, relevant clinical evidence (actual clinical benefit, clinical added value), fulfill data interoperability and security standards requirement.	Yes
Germany	Digitale-Versorgung-Gesetz	CE-marked, low risk patient-facing mobile applications and web-based platforms	Requirement: (1) General requirements (data protection, information security, interoperability, robustness, consumer protection, ease of use, support of healthcare providers, quality of medical service and patient safety), (2) positive healthcare effect (medical benefit, patient-relevant improvement of structure and processes)	Yes
United Kingdom	Evidence Standards Framework Digital Health Technologies DHT classification (Tier A, B, and C) based on the potential risk to service users and to the system. Tier C DHTs are divided into 4 subgroups based on the significance of information and the state of the healthcare situation or condition.	Digital health technologies, such as smartphone apps, standalone softwares, online tools and programs.	21 criteria in five groups: (1) design factors: standards 1–9 (2) describing values: standards 10–13 (3) demonstrating performance: standards 14–16 (4) delivering value: standards 17–18 (5) deployment considerations: standards 19–21	Not mandatory

AI, artificial intelligence; CE marking, Conformité Européenne marking; CMDs, Connected Medical Devices; DHT, Digital Health Technologies; DMDs, Digital Medical Devices; FAMHP, Federal Agency for Medicines and Health Products; GDPR, General Data Protection Regulation; HTA, Health Technology Assessment. Source: Authors own elaboration.

#### 3.2.1. Belgium

The Belgian government founded the mHealthBelgium digital platform in 2018 (29), with a reimbursement framework launched in 2021. The aim of the mHealthBelgium initiative is the integration of software-based health applications in the Belgian health care system. CE-marking is a pre-requisite for assessment and public reimbursement of DHTs in Belgium (29).

To reach public reimbursement, CE-marked software applications should climb three levels of a validation pyramid (8), meeting specific criteria defined by the Belgian government for each level of the pyramid (29), as shown in Supplementary Figure S1. The Federal Agency for Medicines and Health Products (FAMHP) is responsible for managing applications at level M1. To be eligible for level M1 the following criteria should be met by the health software applications: (i) submitted CE declaration, (ii) compliance with the EU General Data Protection Regulation (GDPR) and (iii) voluntary notification to the FAMHP, during which the CE marking and compliance with the rules for medical devices are confirmed (30). Level M2 has specific criteria relating to testing the interoperability and connectivity to the eHealth Platform and is completely managed and supervised by the eHealth Platform. The eHealth Platform is the federal digital health organization responsible for building the healthcare infrastructure for information exchange in Belgium (29). A software application can be eligible for level M2 if there is an independent risk assessment proving that the application meets specific requirement regarding security, authentication, identification, the patient’s therapeutic relationship, informed consent, and the use of local e-health services.

Reaching level M3 of the pyramid can result in temporary or permanent public reimbursement of the solution. The competent authority responsible for reimbursement decisions at this level is the National Institute for Health and Disability Insurance (NIHDI). To pass level M3 for reimbursement, the software application developers must submit a dossier proving clinical and/or socio-economic value the app would bring in the care path (29, 30). At sublevel M3-the solutions are in the process of proving social-economic value, while sublevel M3^+^ grants permanent reimbursement provided that the socio-economic value was fully proven. The apps cannot be financed by themselves, only as part of the health care process (29, 31). This means that by granting reimbursement to an app at level M3 (either temporary or permanent), NIHDI should also reshape the reimbursement of the underlying care paths, which can slow down the implementation of granting reimbursement to applications (8). As of January 2023 there are only two accepted care processes at M3 level, out of which one is a permanently reimbursed application for the treatment and rehabilitation of hip and knee arthroplasty while the second application was temporarily reimbursed for telemonitoring COVID-19 patients, but has been withdrawn since (31).

In Belgium, besides public reimbursement and out-of-pocket payments of patients, hospitals can also propose health software applications to patients via their innovation budget and health insurance companies can also refund at least a part of the usage fee to its insured persons. There is a private health insurance company already providing all listed apps in the mHealthBelgium platform (irrespective of their status in the validation pyramid) to its clients (32).

#### 3.2.2. Finland

The implementation of innovative DHTs, − particularly focusing on AI and robotics, that support smart aging and care at home - was fostered by the publication of Hyteairo and KATI frameworks by the Ministry of Social Affairs and Health in Finland between 2018–2019 (33). Recognizing shortcomings of the KATI framework, a new general-purpose HTA, “Digi-HTA” was developed and implemented in 2019, aimed to cover a broader range of DHTs, by the Finnish Coordinating Center for Health Technology Assessment (FinCCHTA) and University of Oulu (7).

Digi-HTA covers 6 out of 9 domains of a “standard” HTA process, except ethical, social and legal issues. Thus, domains covered are: (1) the health problem and current use of technology; (2) description and technical characteristics of the new technology; (3) safety assessment; (4) clinical effectiveness; (5) economic evaluation, typically cost-effectiveness analysis or cost-utility analysis; (6) organizational aspects. They are left out on purpose allowing for expedited assessments in the rapidly evolving DHT sector. Three documents are used to collect all needed information on the product under assessment from the company. The information on the products under assessment is supplemented by literature reviews carried out by HTA experts and cyber security specialists.

The specific name of domains, − transformed to match DHTs - in Digi-HTA are as follows: (1) product information; (2) technical stability; (3) cost; (4) effectiveness; (5) clinical safety; (6) data security and protection; (7) usability and accessibility; (8) interoperability; (9) patient and organizational considerations; while (10) AI and (11) robotics form two separate segments (34). The most important things in the product recommendation are safety, effectiveness, cost, data security and protection, as well as usability and accessibility. The evidence needed for the assessment is mostly provided by the technology developer, which Digi-HTA typically supplements with literature reviews and expert reviews. The outcome of the Digi-HTA assessment is a traffic light model with the recommendation being valid for 3 years. Reassessment can be requested by the developer in case of significant product/service changes. According to a survey carried out among health professionals and DHT companies, there is a clear need to further integrate into the reimbursement decision-making processes, as there is currently no formal process for this, contrary to Germany (7).

#### 3.2.3. France

In France, the Haute Autorité de Santé (French National Authority for Health - HAS) is the authority for the reimbursement of medical devices. The relevant DHTs from this review’s perspective has been identified as connected medical devices (CMDs) (29). A CMD could be added on HAS’ list of products and services that qualify for reimbursement (Liste des Produits et Prestations Remboursables - LPPR) after considered by Medical Device and Health Technology Evaluation Committee (CNEDiMTS). The National Authority for Health (HAS) published a classification guidance for digital health solutions in 2021. It has no legal/financial consequence but creates a conceptual framework. According to the classification system there are 4 levels (35).

CNEDiMTS assesses CMDs only with fulfilling the following criteria: CE-marked, it is for individual use, there is a telecommunication function and there is a submitted application for reimbursement (32). An updated submission guide published in September 2020 includes CMD specific requirements for the reimbursement dossier in which the following evidence defined to be assessed: actual clinical benefit, clinical added value, intended role in the therapeutic strategy for a given disease, indications, usage and target population. CNEDiMTS also takes into account the severity of the disease, efficacy and adverse effects of CMD, intended role in the therapeutic strategy in comparison to other available therapies and public health benefits (36, 37). Proven clinical added value has an impact on the reimbursed price of CMD negotiated by the French Healthcare Products Pricing Committee. Reimbursement is granted for a maximum of 5 years (32).

Besides to the centralized pathway of reimbursement listing, defined health apps can also be reimbursed for telemonitoring via the experimental program called ETAPES (Expérimentation de Télémédecine pour l’Amélioration des Parcours en Santé). It has been a good pathway to obtain coverage for telemonitoring apps in one of the following indications: heart failure, kidney failure, respiratory failure, diabetes, and implantable cardiac devices. The provided funding might be payment for the medical professional performing telemonitoring or therapeutic support to the patient, or payment to the provider providing technical solution for telemonitoring (37, 38).

Under the impact of the COVID-19 epidemic, the French Ministry of Health announced that telemonitoring would be reimbursed through general legislation. Five telemedicine services have been defined provided by medical health professional as follows: (1) teleconsultation; (2) consultation via ICT; (3) telesurveillance - monitoring patient data via ICT; (4) tele-expertise - solicit the advice of one or more medical professional colleagues remotely through ICT; (5) teleassistance – a medical health professional remotely assist another health professional; (6) medical regulation - activity of the centers for emergencies. Neither teleassistance nor medical regulation responses are subject to specific market access procedures as no patient included. As of 2020 certain remote cares are available as telemedicine services provided by pharmacists and paramedical professionals (32).

In October 2021 an accelerate digital health strategy has been introduced with the intent to simplify market access for digital health solutions. The launched “fast track” reimbursement mechanism modeled on the DiGA mechanism in Germany. Note that the term CMD has been replaced by digital medical device (DMD) in the most recently published documentations. Fast track preliminary access is applicable for DMDs which: already CE marked, provide digital therapeutics and innovative telemonitoring solutions with clinical evidence and fulfil data interoperability and security standards requirement. Within this accelerated procedure DMDs gain 1 year to apply for normal reimbursement pathway (34). Authorities have published a number of guidelines to facilitate the uptake of DMDs into the French health care system.

#### 3.2.4. Germany

The German Digital Healthcare Act (“Digitale-Versorgung-Gesetz”) was enacted in 2019 aiming to increase the adoption in the German healthcare system of high-quality “DiGAs” (“Digitale GesundheitsAnwendungen”), as part of the therapeutic process across several disease indications (39). The German Federal Institute for Drugs and Medical Devices (BfArM) defines DiGAs as mobile applications or web-based platforms that are class I or IIa medical devices (already CE-marked) with a therapeutic purpose (primary prevention excluded) and aimed at patients and not healthcare professionals (e.g., telemonitoring technology that acts as decision support for physicians are excluded) (36).

The innovative regulation introduces a Fast-Track procedure to evaluate and reimburse digital health apps. Even if the positive healthcare effect of the DiGA is not yet established, a preliminary admission into the directory of reimbursable DiGA can be granted. BfArM grants a permanent listing of the DiGA in the directory if sufficient scientific evidence of a positive healthcare effect is demonstrated along the lines of patient-reported outcome measures as well as endpoints related to medical outcomes and healthcare experience. If only provisional listing is granted, the manufacturer has 12 months to complete the clinical trial(s) required by BfArM and demonstrate a positive healthcare effect of the technology. Both in case of a provisional or permanent listing of a technology in the DiGA directory, the DIGA is covered by statutory health insurance which gives access to innovative digital health solutions to more than 76 million German citizens insured under the statutory health insurance scheme.

The evaluation criteria is drawn up in two major categories; (1) General criteria, including security, functionality, quality, patient safety, interoperability, data security amongst others and (2) positive healthcare effects. Positive care effects are defined either as (a) a medical benefit (e.g., an improved health status or a shorter disease duration) or (b) a patient-relevant improvement of structure and processes (e.g., improved patient autonomy, increase in health literacy, facilitated access to care or disease coping strategies). To prove a DiGA’s positive healthcare effect, manufacturers must provide quantitative results of a comparative study conducted in Germany as part of their application. This study needs to demonstrate the superiority of using the DiGA over not using it in terms of at least one claimed positive healthcare effect (9). The gold standard study design to prove a positive effect is a randomized control trial, however they also accept alternative study designs like Pragmatic Clinical Trials (PCT), Sequential Multiple Assignment Randomized Trial (SMART) or Multiphase Optimization Strategy (MOST), with the minimum requirement being a retrospective comparison (40). Despite accepting evidence based on alternative research studies, the majority of study designs of accepted DiGAs are based on randomized controlled trials (RCTs) with a focus on medical benefits.

As of March 2023, 45 DiGAs are available on prescription in Germany, of which 17 technologies have been permanently listed in the DiGA directory (41). Currently reimbursed digital apps and web-based platforms support patients in the areas of mental health (depression, anxiety, stress, panic disorder), diabetes, musculoskeletal disorders and smoking cessation among others. The DiGAs cost between €200 and €740 in a subscription model for a 90-day period.

The German authorities are considering introducing similar mechanisms for digital care applications or DiPAs (Digitale Pflegeanwendungen) to assist citizens in care facilities, and to expand the scope of DiGAs to higher class digital applications (DiGAs class IIb and III). The scope may further be widened to non-patient facing digital health solutions (e.g., clinical decision support tools) or to include apps used at the hospital level to reduce costs and drive patient outcomes (29).

#### 3.2.5. United Kingdom

The National Institute for Healthcare and Excellence (NICE) was the first HTA body globally to publish an evidence standards framework for digital health technologies in 2018, which since then has been regularly revised and updated. The framework determines standards for DHTs to demonstrate their value in the healthcare and social care system. The classification of DHTs (Tier A, B and C) is based on the potential risk to service users and the system (42). Tier A includes DHTs providing system services that intend to save time or costs but do not have direct patient health or care outcomes. Tier B includes DHTs (such as health and care diaries or health promotion apps) that help users to manage their health. Tier C category includes four subcategories of DHTs based on the framework of the International Medical Device Regulators Forum: for diagnosing or treating a specific condition or informing/driving clinical management (43). Tier C DHTs are technologies directly affecting health outcomes of the user; therefore, they must meet more stringent qualifications than Tier A and B technologies.

The framework is used to evaluate DHTs such as phone apps, standalone software and tools that support data analysis, the detection and treatment of conditions, or care management. The framework does not apply to software in medical devices and DHTs that enable data management in healthcare research and training of healthcare workers (44).

It sets out 21 criteria for assessing DHTs, divided into five groups. Within the (1) “design factors” group, there are nine standards, such as compliance with relevant quality and safety standards, describing processes of creating reliable data and information, proving environmental sustainability and considering inequalities and bias mitigation. Within the (2) “describing value” group, four standards are required to describe the target population, the intended purpose, proposed pathways or processes, and the expected costs and health outcomes compared with current processes. In the (3) “demonstrating performance” group, it is necessary to provide real-world evidence for benefits that can be realized in practice and to provide a plan for monitoring changes in the use and performance of DHTs. Developers of Tier C technologies also need to demonstrate the effectiveness of the DHTs. A budget impact analysis and cost-effectiveness analysis are required in the (4) “delivering value” group. Finally, in the (5) “deployment considerations” group, developers must provide transparent deployment requirements, scalability, and clear user guidance.

The NICE framework identifies real-world evidence, observational studies, expert opinions, or evidence synthesis studies as the source of clinical evidence for “Tier A and B” DHTs. For “Tier C” DHTs, test accuracy studies, concordance studies, interventional studies, retrospective studies, or prospective studies are identified as possible sources of clinical evidence. Qualitative studies on patient or medical professional experience are also accepted for “Tier C” DHTs. Although the Evidence Standards Framework for Digital Health Technologies was defined by NICE, but it is left to Clinical Commissioning Groups and regional National Health Service Trusts to negotiate reimbursement with developers, and the use of the framework is not yet mandatory in the public reimbursement process for DHTs (10).

### 3.3. HTA and public financing of digital health technologies in Hungary

HTA methodologies in Hungary were partially implemented in 2004, including cost-effectiveness analysis to support pricing and reimbursement decisions regarding new medicines (14, 15). Public funding of health technologies is regulated by law in Hungary, the decision maker is the public payer; the National Health Insurance Fund (NEAK). For a social security subsidy health economic analysis in the application dossier must be prepared in accordance with the national health economic guidelines. The guideline for the preparation and evaluation of health economic analyses was published in 2021 November and is valid until November 2024 (45). Currently, neither the legal framework governing submissions nor the HTA guide address DHTs in Hungary. However, there are no obstacles to the submission/evaluation of these technologies, as long as they comply with the legislation and the HTA guideline in force.

Despite a lack of HTA framework for DHTs, some telemedicine procedures have received coverage in 2020. Act 58 of 2020 defined the concept of telemedicine to ensure the continuity of patient care (46), when no in-person health care was provided with the exception of COVID-19 and some defined cases during the COVID pandemic. Under the Decree, a physician or a healthcare worker may, within his/her competence, without personal presence, provide care in the outpatient settings enlisted in Table 2. The specified telemedicine services are funded with a given fee parameter. While the provision was in force temporary set to end in September 2020, it has since been made permanent. According to the explanatory memorandum of the Decree, telemedicine reimbursement is only the first element of a comprehensive regulation that allows telemedical solutions to be widely used by providing a legal basis for it. It needs to be emphasized that the Hungarian telemedicine reimbursement Act is not equivalent to the international assessment frameworks explored above, as it does not base the reimbursement decision on certification or health technology assessment of the telemedical solution.

**Table 2:** Covered telemedicine activities in Hungary.

Professional assessment of the patient’s state of health
Detecting diseases and their risks
Identification of the specific disease(s)
Order further tests to assess the patient’s condition more accurately, and start treatment
Teleconsultancy (establishing the effectiveness of treatments)
Monitoring the patient’s condition and making a diagnosis based on ICT
Psychotherapy, crisis intervention, parent consultation, counseling, supportive psychotherapy
Physiotherapy using a remote consultation device
Breastfeeding counseling
Telephone, online or other forms of advice and consultation

Source: (46).

There are no special provisions about the reimbursement and use of digital health apps or web-based platforms. This means that health apps classified as medical devices based on the European Medical Devices Regulation (MDR) and have a CE-mark cannot be officially reimbursed by national health insurance fund. Thus, there is no division between DHTs compliant with MDR or with demonstrated clinical benefits and the wide range of wellness apps that are on the market. Technology developers aiming for traction in the Hungarian market focus on payment models like out-of-pocket payments or co-operations with private healthcare providers. This not only creates a gap in accessibility to DHTs for all patients to benefit those who can afford private health services but also leads to the majority of technology developers targeting foreign markets before establishing a Hungarian market presence.

### 3.4. Recommendations for Hungary

Based on a European outlook and the current Hungarian circumstances, the Digital Health working group of the Hungarian Health Economic Society proposes the following actions to support uptake of evidence-based digital health technologies in Hungary:

1. Map and understand the healthcare decision-makers’ information needs about DHTs and the barriers to utilizing them.
2. In lack of a harmonized DHT HTA framework on European level, develop a framework jointly with the National Institute of Pharmacy and Nutrition and the State Department of Health by adopting a pioneer country’s classification, assessment framework and methodological guidelines with relevant adjustments to the Hungarian socio-economic, geographical conditions.
3. Support the European Taskforce for Harmonized Evaluation of Digital Medical Devices effort to shape consensus on what DHTs are and how their clinical benefits should be assessed. Once there is consensus on harmonized assessment criteria across Europe, aim to tailor the existing framework to the consensus criteria.
4. Identify indications/priority areas of public health importance for which there is a demonstrated role for DHTs and where adopting technology can lead to significant social and economic gains. Different groups can be identified with therapeutic equivalence, which do not need to be fully re-evaluated if similar or equivalent safety and efficacy parameters shown as in the case of comparator.
5. Consider a conformity assessment procedure for DHTs as a medical device prior to HTA. CE certification of DHTs prior to HTA demonstrates the overall safety and performance of the medical device. The majority of European countries have already moved toward making it a pre-requirement before assessing and potentially reimbursing DHTs.
6. Describe benefit(s) for the end user, demonstrate why the DHT is innovative and to what extent it is superior (what is the added value) to the standard of care. It is valuable to define superiority, as a technology may be superior if it is more expensive but realizes more health gains. However, it may also be superior if it realizes the same health gains but is cheaper because it replaces significant human resource capacity or improves health outcomes due to improved adherence. In resource-limited countries, it may be appropriate to prefer DHTs that fall into the latter group, i.e., allowing cost savings.
7. Introduce a publicly accessible directory for certified, trusted and safe digital health solutions that are CE-marked and comply with data protection regulations (e.g., based on European Database on Medical Devices (EUDAMED) or Belgian mhealth pyramid) to support alternative models of financing, e.g., out-of-pocket payment, co-operations with private health care organizations and private health insurance, public procurement.
8. Create a network of digital health “Living Labs/Testing Facilites” that offer sustainable and real-life environment to ideate, develop, test, and validate digital health innovations with the involvement of academia, innovators, healthcare providers and public institutions responsible for health policy, including the State Department of Health.

The above recommendations should also be considered by other countries with similar economic status and healthcare governance.

## 4. Discussion

In an effort to better integrate digital health solutions in day-to-day care, a number of countries have begun to adapt their national frameworks and policies in recent years. With almost as many approaches to this as health systems in Europe, significant differences can be observed in the types of technologies countries have included, as well as the methods chosen to assess and make solutions available to patients. German DiGAs so far are mostly low-risk health apps and web-based platforms, whereas other countries like France and Finland are also integrating telemedicine, AI solutions, and robotics in their frameworks (7, 10, 11). There are also considerable differences in the assessment domains across the studied countries, with the outcome of the assessment ranging from a multi-level tier system, traffic light system or simple go/no-go decisions. Depending on the given country DHTs are partly paid voluntarily by patients and individual health insurers, or individual DiHA with certain proof of patient benefit are paid obligatorily by all health insurers (e.g., in Germany, Belgium, France) (11).

Each country has its own specific needs related to the implementation of DHTs determined by several factors: technical infrastructure, openness to innovation, digital health literacy, available budget resources, health priorities, culture, etc. The German DIGA, the French DIHA, the Belgian mHealth, and the British ESF and the Finnish Digi-HTA – with their advantages and disadvantages - can be exemplary in determining the extent to which the appraisal rules should be modified in order to be inclusive of digital health solutions. However, allowing the multiplication of different frameworks for market access of DHTs across Europe not only risks leaving behind entire patient populations in late adopting, but it also threatens by making the European market unpracticable for digital health technology developers and innovators. Fragmentation of this field would eventually lead to Europe losing momentum in becoming a primary player in digital health care with EU-based innovators targeting the US and Asian markets before establishing a European market presence.

With the common goal of harmonizing evaluation procedures for DHTs across Europe, a European Taskforce was established under the French Presidency of the Council of the EU with the coordination of EIT Health (47). The Taskforce for Harmonized Evaluation of Digital Medical Devices (DMDs) brings together pan-European experts from nine EU Member States with the aim of harmonizing the nomenclature and taxonomy of DMDs based on their application purpose, reaching a consensus on evidence requirements in the light of national implementation requirements and recommend on a health system implementation framework The taskforce seeks to advise the HTA Coordination Group (HTAR), national responsible authorities and agencies, innovators and policymakers – in alignment with EU medical device regulators – on the development of a joint DMD assessment procedure, including the definition of DMDs based on their application purpose and evaluation categories.

Similar to “traditional” medical devices, patient safety aspects are of paramount importance, following the ancient Greek expression of “first do no harm.” As of current knowledge, there is evidence that digital improvements to existing technologies can reduce harm, but new technology and processes are always a risk factor for errors, as they increase burden on staff as the learning curve can be steep. A study looking at 10 years of health information technology failures found that incidents involving digital technologies are preventable in 75% of cases, with adequate staff training, and collaboration with clinicians during product development (48–51). Every framework used to evaluate DHTs should contain patient safety aspect as first considerations. Evidence is already available from UK that digital tools can be effective in reducing medication errors, as demonstrated by the PINCER – study, led by pharmacist (52).

The MDR and CE-mark are insufficient as a framework for developing reliable digital health innovations. Therefore, more focus on Health Technology Assessment guidelines specific to DHTs is needed. As the majority of assessment frameworks are based on solutions being CE-marked under the MDR, they could theoretically serve as a model for introducing health applications on prescription in other EU Member States (50). Once digital technologies are CE marked, joint HTAs similar to those already conducted in networks like the EUnetHTA could be an initial way of establishing benefit and provide a basis for reimbursement conditions to be specified in the countries. The 2021/2282 EU regulation on health technology assessment establishes a framework for joint clinical assessments of new medicines and certain high-risk medical devices (53). However, this excludes most digital health technologies, which typically fall into lower risk categories. The impact of other regulations including the proposal on the European Health Data Space, Data Act or the Artificial Intelligence Act are equally important but remain unclear.

The study has the following limitations. Our literature review did not cover all relevant countries’ financing and HTA practices of DHTs. We only looked at a few pioneering EU countries, but several non-European countries are also at an advanced stage of regulating DHTs such as USA, Canada, Australia, South Korea. As the field under study is evolving quite dynamically, the individual systems may look different at the time of publication of this article compared to the manuscript. Nevertheless, we have tried to balance the breadth of the scope of the subject and the depth of the analysis.

## 5. Conclusion

There is uncertainty surrounding the directions that individual countries will take in the coming years, but what the above country examples unequivocally show is that digital health solutions have gained recognition in recent years and are increasingly being evaluated and funded in many parts of Europe. This presents health authorities with novel challenges around assessment and implementation of technologies that outpace traditional timelines in their development, overwhelm the capabilities of national institutions with their numbers, and transcend existing reimbursement models in the value they bring to patients. Having mapped the European health technology assessment and reimbursement landscape, this paper offers an outline of recommendations for country-level action that relevant stakeholders can and should consider in order to support uptake of digital health solutions in Hungary.

While each country has its own specific needs related to the implementation of DHTs, the Digital Health working group of the Hungarian Health Economic Society recommends for Hungary to develop a framework for market access for DHTs by adopting a pioneer country’s assessment framework with relevant adjustments to the Hungarian socio-economic, geographical conditions, as a harmonized European framework is not yet available. It is recommended to identify priority areas of public health importance for which there is a demonstrated role for DHTs and where significant social and economic gains can be achieved by adopting digital health technologies. In a resource-limited country, it may be appropriate to prioritize DHTs that offer cost savings, for example, by realizing the same health gains as a comparator but at a lower cost by replacing significant human resource capacity. It is clear that the specificities of national healthcare contexts must be taken into account in decisions to allocate public funds to certain therapies rather than others and therefore countries should be actively taking part in shaping common clinical evidence standards and technical quality criteria in order for common standards to be implemented in a way that supports all European Members States.

## Author contributions

FM: conceptualization. FM and GT: methodology. FM, KH, MP, KL, IA, and GT: writing – original draft preparation. FM, KH, and GT: writing – review and editing. All authors listed have made a substantial, direct, and intellectual contribution to the work and approved it for publication.

## Funding

The study was funded by the National Research, Development and Innovation Office in Hungary (RRF-2.3.1-21-2022-00006, Data-Driven Health Division of National Laboratory for Health Security). Project no. KDP-14-3/PALY-2021 has been implemented with the support provided by the Ministry of Culture and Innovation of Hungary from the National Research, Development and Innovation Fund, financed under the KDP-2020 funding scheme under grant agreement N°1012597.

## Conflict of interest

KL was employed partly by CE Certiso Ltd.

The remaining authors declare that the research was conducted in the absence of any commercial or financial relationships that could be construed as a potential conflict of interest.

## Publisher’s note

All claims expressed in this article are solely those of the authors and do not necessarily represent those of their affiliated organizations, or those of the publisher, the editors and the reviewers. Any product that may be evaluated in this article, or claim that may be made by its manufacturer, is not guaranteed or endorsed by the publisher.
