# Supplementary material for: International practices in health technology assessment and public financing of digital health technologies: recommendations for Hungary
Source: Front Public Health. 2023 Aug 31;11:1197949. doi: 10.3389/fpubh.2023.1197949 (PMC10501404; doi:10.3389/fpubh.2023.1197949)
Supplement: Supplementary file 1 [file Data_Sheet_1.docx]

***Supplementary Material***

International practices in the public financing of digital health technologies: implications for Hungary

# Supplementary Figure 1 The levels of the validation pyramid in Belgium


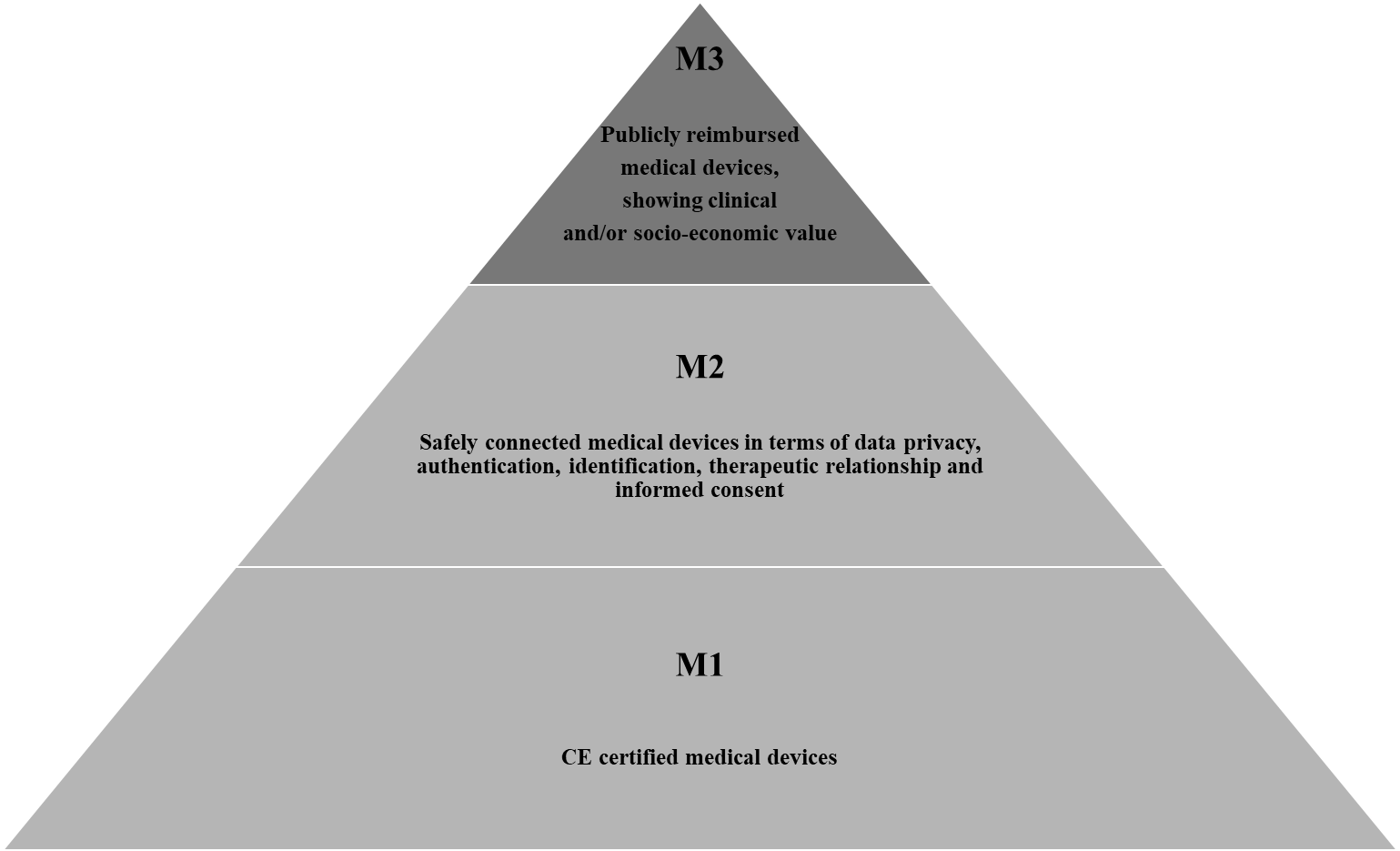


# Source: (1)

**References**

1. Medtech Europe. Recognising the value of digital health apps: An assessment of five European healthcare systems. (2021). Available online: https://www.medtecheurope.org/wp-content/uploads/2021/11/2111_v4.8_mte_dht_reimbursement16.11.2021-2.pdf (accessed January 31, 2023)
